# Evaluation of CAMP-Like Effect, Biofilm Formation, and Discrimination of* Candida africana* from Vaginal* Candida albicans* Species

**DOI:** 10.1155/2017/7126258

**Published:** 2017-11-26

**Authors:** Keyvan Pakshir, Mahboubeh Bordbar, Kamiar Zomorodian, Hasti Nouraei, Hossein Khodadadi

**Affiliations:** ^1^Basic Sciences in Infectious Diseases Research Center, Department of Parasitology and Mycology, School of Medicine, Shiraz University of Medical Sciences, Shiraz, Iran; ^2^Department of Parasitology and Mycology, School of Medicine, Shiraz University of Medical Sciences, Shiraz, Iran

## Abstract

*Candida africana as* a species recovered from female genital specimens is highly close to* C. albicans*. The present study was conducted to discriminate* C. africana* from presumptive vaginal* C. albicans* strains by molecular assay and evaluate their hemolysin activity, biofilm formation, and cohemolytic effect (CAMP) with vaginal bacterial flora. A total of 110 stock vaginal* C. albicans* isolates were examined by* HWP1* gene amplification. Hemolysin activity and the ability of biofilm formation were evaluated by blood plate assay and visual detection methods, respectively.* Staphylococcus aureus*,* Staphylococcus epidermidis*, and* Streptococcus agalactiae* were used to evaluate the CAMP-like effects in Sabouraud blood agar media. Based on the size of the amplicons (941 bp), all isolates were identified as* C. albicans*. All samples were able to produce beta-hemolysin. Moreover, 69 out of 110 of the isolates (62.7%) were biofilm-positive, 54 out of 110* Candida* isolates (49%) demonstrated cohemolytic effects with* S. agalactiae*, and 48 out of 110 showed this effect with* S. aureus* (43.6%). All isolates were CAMP-negative with* S. epidermidis*. We detected all isolates as* Candida albicans* and almost half of the isolates were CAMP-positive with* S. aureus* and* S. agalactiae*, suggesting that these bacteria increase the pathogenicity of* Candida* in vaginal candidiasis.

## 1. Introduction

*Candida albicans *is a normal constituent of the human flora present in vaginal mucosa and gastrointestinal tract. This species is the most common fungal pathogen isolated from patients with vulvovaginal candidiasis (VVC) [1]. More recently, atypical strains of* C. albicans *such as* Candida africana, *closely related to* C. albicans and Candida dubliniensis, *have been reported as the cause of vaginitis [2].* Candida africana *has been isolated for the first time from patients in Africa and Germany and studied by Tietz et al. [3]. It also has been reported as a cause of VVC in many countries [4–6]. Romeo and Criseo described a specific molecular method for differentiating* C. albicans*,* C. africana*, and* C. dubliniensis, *which involves the use of a single pair of primers targeting the hyphal wall protein 1* (HWP1)* gene and its homologs in a polymerase chain reaction- (PCR-) based assay [7]. Hyphal wall protein 1 is a gene that is required for virulence in systemic candidiasis [8]. Several factors such as adhesion, the formation of biofilm and germ tube, phenotypic switching, and synergy with bacteria and production of hydrolases have been proposed to trigger* Candida *spp. virulence [9].

Biofilms play an important role as reservoirs for pathogenic agents, allow coinfection with other pathogens, promote persistence of infection, and increase mortality rate. The ability of* Candida* species to form biofilms is an important factor in pathogenesis [10, 11]. Hemolysins play an important role in the infectious process in fungal and bacterial infections and have been reported to have cytotoxic, pore-forming, and lysis effects on eukaryotic cells [11]. The hemolytic factor of* Candida* species has been described by many researchers and a hemolysis test was developed to determine hemolytic factor [12, 13]. Correlation between fungi and bacteria sometimes leads to synergism in their pathogenicity. The cohemolytic effect, also known as “CAMP reaction,” was first described by Christie, Atkins, and Munch-Peterson in 1944. The cooperative (CAMP-like) lytic processes result from the interaction of at least two membrane-active agents of bacteria, with biological membranes [14]. The vaginal microbiome plays an important role in female health and consists of yeasts and different bacteria species.

Group B streptococci (GBS) are bacterial species that colonize the vagina in pregnant women.* Streptococcus agalactiae* is a commensal bacterium of human gastrointestinal and genital tracts. Recent studies have shown asymptomatic colonization rates of up to 36% in healthy women [15, 16].* Staphylococcus epidermidis* and* Staphylococcus aureus* are two other bacteria that could be found in general flora of the female genital tract [17, 18].

To the best of our knowledge, there are no data on the cohemolytic effect of bacteria species that are generally part of normal vaginal flora with* Candida albicans* species causing vaginal candidiasis. In our previous studies, by using conventional and PCR-RFLP methods, no* Candida africana* was identified as the causative agent of vulvovaginal candidiasis and less data were found about the pathogenicity of the isolates [19, 20]. In the present study, we reexamine vaginal* Candida spp* isolates presumptively identified as* Candida albicans* by primary screening tests (germ tube positive and green color colony on CHROMagar media) for the presence of* C. africana* based on* HWP1* gene amplification. Moreover, the ability of biofilm formation and hemolysin activity of the isolates are measured and, to determine synergism with bacteria, the cohemolytic effect (CAMP-like phenomenon) of the isolates with* Staphylococcus aureus, Staphylococcus epidermidis,* and* Streptococcus agalactiae* were evaluated.

## 2. Material and Methods

### 2.1. The Isolates

A total of 110 stock strains of* Candida albicans* species recovered from patients with vaginal candidiasis were included in this study. All species were identified as* Candida albicans*, mostly based on initial screening through use of germ tube test and a green color colony on chromogenic medium (CHROMagar* Candida*, France). The isolates were subcultured on Sabouraud dextrose agar (Merck, Germany) and incubated at 30°C 24 h before use.

### 2.2. *HWP1* Gene Amplification

Genomic DNA from* Candida* isolates was extracted through the boiling method described by Makimura et al. [21] with small modifications. Briefly, a small amount of yeast colony was boiled for 15 minutes in lysis buffer containing 30 mM EDTA, 0.5% SDS, and 100 mM Tris-HCl. A solution of potassium acetate (2.5 M) was then added to the lysate and held on ice for 1 hour and then centrifuged at 12000 rpm for 5 min and the supernatant was transferred to a new tube. Yeast DNA in the supernatant was precipitated with isopropanol, washed twice with ethanol, air-dried, and resuspended in 50 *μ*l of distilled water prior to use for PCR.

The PCR primers (CR-f 5′-GCTACCACTTCAGAATCATCATC-3′ and CR-r 5′-GCACCTTCAGTCGTAGAGACG-3′) were used for* HWP1* gene amplification to distinguish among* C. albicans*,* C. dubliniensis,* and* C. africana* based on the distinct amplicon size, 700 bp for* C. africana*, 569 bp for* C. dubliniensis*, and approximately 941 bp for* C. albicans.* Amplification was carried out in a final volume of 25 *μ*l using Ampliqon master mix kit (Denmark). The PCR reaction conditions for 35 cycles were as follows: initial denaturation at 94°C for 5 min, denaturation step 30 s at 94°C, annealing at 62°C for 45 sec, and extension for 45 sec at 72°C, followed by a final extension at 72°C for 7 min. PCR products were separated in a 1.3% (wt/vol) agarose after gel electrophoresis and visualized by staining with ethidium bromide (0.5 *μ*g/ml).* C. albicans* (ATCC 10261),* C. dubliniensis* (CBS 8501), and* C. africana* (IFRC 707) were used as controls.

### 2.3. Assessment of Hemolytic and CAMP-Like Effect

Hemolytic activity was evaluated using blood plate assay described by Luo et al. [22]. Sabouraud dextrose agar of sheep blood was prepared by adding 7% v/v of fresh sheep blood and 3% w/v glucose. A yeast suspension equivalent to 2 McFarland turbidity standards was prepared using the pure yeast cultures. Then, 10 *μ*L of inoculation was spotted on sheep blood SDA plates and incubated at 37°C for 48 h. The presence of a distinctive translucent halo around the inoculum site indicated positive hemolytic activity. The results were expressed as beta (complete), gamma (incomplete), and alpha (no hemolysis). Evaluation of cohemolytic effect was performed using standard strains of bacteria including* Staphylococcus aureus* (ATCC25923),* Staphylococcus saprophyticus* (PTCC1440), and* Streptococcus agalactiae* (PTCC1768). After 2 days of incubation for the evaluation of hemolytic activity, a loop was used to streak each bacterium in straight lines across the plate at a distance of 10 mm from the edge of yeast colony border. The plates were then incubated at 37°C and inspected daily for 2 days. A distinct arrowhead of hemolysis at the intersection of the tester strain and the* Candida* colony streaks was considered as CAMP positive [21].* C. albicans* (ATCC10261) was used as a positive control.

### 2.4. Determination of Biofilm Formation

The evaluation of biofilm formation was carried out through the visual detection (tube method) [10]. Briefly, 10 mL of Sabouraud dextrose broth medium supplemented with glucose (final concentration 8% w/v) in a screw-capped conical polystyrene tube was inoculated with loopful of yeast colonies and incubated without agitation at 35°C for 48 h. After incubation, the broth in the tube was aspirated gently using a sterile Pasteur pipette. Tubes were washed twice with sterile distilled water and then stained with 1% (w/v) safranine. Next, the tubes were decanted after 10 min and examined for the presence of an adherent visible biofilm layer at the bottom and on the wall of the tubes. The test was run in duplicate and the results were expressed as negative (−), weak (+), moderate (++), and strong (+++).* Staphylococcus epidermidis* (PTCC 1435) and* C. albicans* (ATCC10261) were used as positive controls.

### 2.5. Statistical Analysis

The Chi-square and Fisher's exact tests were used for data statistical analysis. A *P* value ≤ 0.05 was considered as significant.

## 3. Results

**Figure 1:**
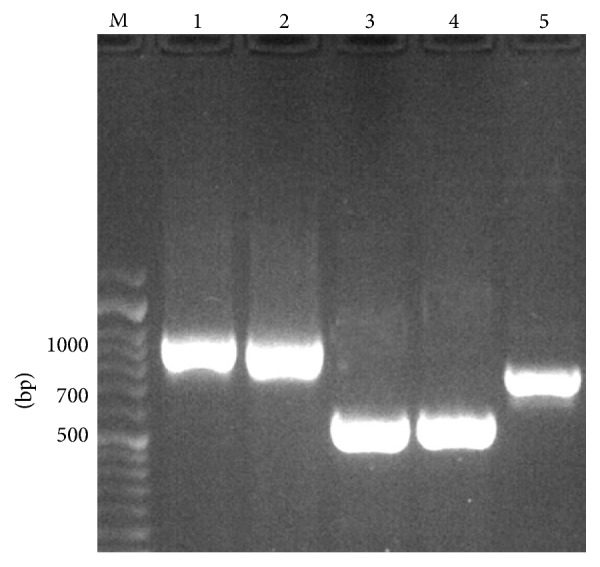
Agarose gel electrophoresis of* HWP1* gene amplification (PCR products):* C. albicans* (ATCC10261) lane 1,* C. albicans* isolate (lanes 2),* C. dubliniensis *(CBS 8501) lanes 3 and 4,* C. africana* (IFRC707) lane 5, and molecular size markers lane M.

In this study, 110 presumptive strains of vaginal* Candida albicans* were examined for the presence of* Candida africana* by* HWP1* gene amplification. All the isolates produced a DNA fragment of 941 bp, indicating that these strains belong to* C. albicans*. Also, none of the isolates produced a DNA fragment of 700 bp, corresponding to* C. africana*, or 569 bp fragment corresponding to* C. dubliniensis* (Figure 1).

Blood plate assay reveals that all the isolates had hemolytic activity and exhibited the zone of complete hemolysis (beta) on sheep blood SDA at 48 h after inoculation: 41 (37.3%)* C. albicans* strains were biofilm-negative, 22 (20%) of the strains were strongly positive, 19 (17.3%) strains were moderately positive, and 28 strains (25.4%) were weakly positive (Table 1).

**Figure 2:**
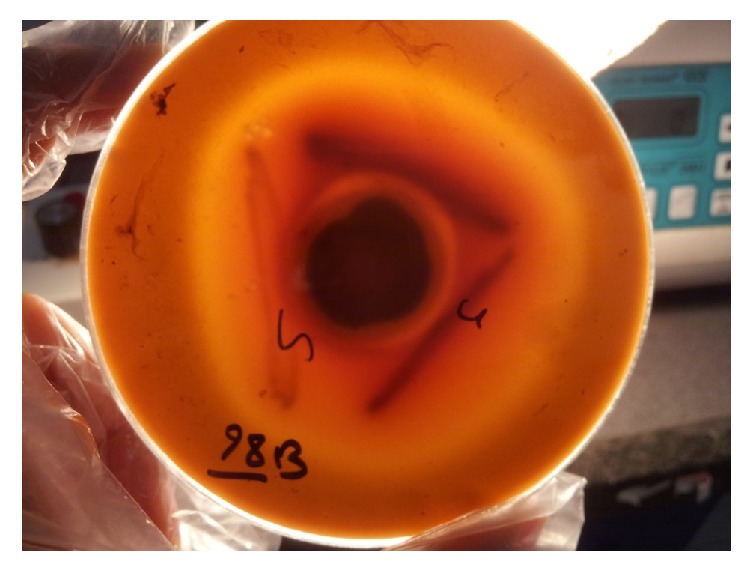
Cohemolytic effect: CAMP-positive (zone of half-moon shaped hemolysis) with* S. aureus* and* S. agalactiae*. CAMP-negative (left) with* S. saprophyticus*.

The CAMP phenomenon was detected using* Staphylococcus aureus* and* Streptococcus agalactiae*. The results show that 54 out of 110 (49%) and 48 out of 110 (43.6%) of the isolates produced a zone of complete half-moon-shaped hemolysis below the border of fungal colony and colony streak of* S. aureus* and* S. agalactiae*, respectively (Figure 2). The remaining strains failed to produce a positive result.

No cohemolytic effect was detected using* Staphylococcus saprophyticus* (Table 1).

## 4. Discussion

Vulvovaginal candidiasis (VVC) is one of the most common forms of* Candida* infections, mostly caused by* Candida albicans*, which affect up to 75% of women at least once in their lifetime [23].* Candida africana* is an opportunistic yeast pathogen that has been linked to vaginal candidiasis. This yeast was first described in 1995 as an atypical chlamydospore-negative* Candida albicans* strain [24].

For the past 15 years, several reports have described “atypical” isolates of* C. albicans* and demonstrated that* C. africana* and* C. albicans* isolates are too closely together. Also,* Candida africana* has been considered as a new species based on ribosomal DNA sequences data in phylogenetic studies. It is often difficult to identify such atypical* Candida* strains at the species level by routine tests [2, 3]. Romeo and Criseo [7] described a specific molecular method for differentiating* C. africana* from* C. albicans*, using a single pair of primers targeting the* HWP1* gene. By this method, Yazdanparast et al. could identify five isolates (4.38%) as* C. africana* from Iranian patients with vulvovaginal candidiasis [6]. Similar to our results, Gumral et al. [25] in Turkey could not identify any* C. africana* species among vaginal* C. albicans* isolates.

Shan et al. in China among 1014 isolates presumptively identified as* C. albicans* could identify 15 (1.5%) to be* C. africana* isolates [1]. In their study, none of the isolates was verified to be* C. dubliniensis*, which is in line with our study. Mucci et al. in Argentina [26] by amplification of the* HWP1* gene on 42* C. albicans* isolates could not identify any isolates of* C. africana*. In another work, Hazirolan et al. [27] in Turkey, among 375 vulvovaginal* C. albicans* complex species, could discriminate only three isolates (0.8%) of* C. africana* by different molecular methods (*HWP1*, ITS, and D1/D2).

These data indicated the low prevalence of* C. africana* in vulvovaginal samples. This low rate is mostly because* C. africana* have an atypical carbohydrate assimilation profile and cannot be identified from* C. albicans* complex species by routine laboratory tests [27]. Moreover, since molecular methods are not used for diagnosis of this species in common laboratories, the results are often misdiagnosed. Despite worldwide distribution of* C. africana*, due to the low incidence rate, it is necessary to study more samples. Biofilm formation is one of the major factors contributing to the virulence of* Candida*.* Candida* cells exhibit distinct properties in a biofilm such as resistance to several antimicrobial drugs [9]. The range of positivity in biofilm formation of* C. albicans* species had variation in different levels in many study reports [9, 10, 28]. The results of the present study reveal that 62.7% of isolates were biofilm-positive and only 20% of those demonstrate strong biofilm formation based on our categories. Yigit et al. in Turkey [9] reported that 88.0% of* C. albicans* strains were biofilm positive and, in Mukhia et al. study [28], 52.3% of* C. albicans* isolates were biofilm-positive. Overall, we could consider that this factor has a potential for pathogenicity.

The capacity of* Candida* spp. to colonize host tissue and cause tissue invasion has been associated with its ability to produce extracellular enzymes [9]. The hemolytic factor of* Candida* species as a probable virulence factor of pathogenesis was described by many investigators and, in most studies,* C. albicans* exhibit beta hemolytic activity [11–13, 29]. All the isolates in our study were able to produce beta-hemolysin. Thus, we could suggest that this factor has a role in pathogenesis on the isolates. The first step in the pathogenesis of vaginal infections is the interaction of the microbial agents with the normal vaginal microflora.

Cooperative (CAMP-like) lytic processes are the result of the interaction of at least two membrane-active factors of bacteria with biological membranes.

There are limited data on CAMP-like activities on dermatophyte pathogenesis, but not on* Candida* spp. [30, 31]. It is therefore important to investigate the possibility of cooperative hemolytic activities between* Candida* and vaginal bacterial microflora. The vaginal microflora, which is composed of a different type of bacteria, presents one of the most important defense mechanisms for preventing the proliferation of microorganisms' foreign to the vagina.* Staphylococcus aureus*,* Staphylococcus saprophyticus,* and* Streptococcus agalactiae* are bacteria that could be found occasionally or permanently as vaginal microflora [17, 18]. Our data indicate that almost half of the vaginal* Candida albicans* isolates in this study had synergy with* S. aureus* and* S. agalactiae*, but not with* S. saprophyticus.* Our data show that existence of these bacteria in the vaginal area and close to* Candida spp* can increase the pathogenesis of* Candida* spp.

## 5. Conclusion

Despite global and broad geographical distribution of* C. africana,* we could not isolate any species of this organism from the vaginal presumptive isolates of* Candida albicans*. By amplification of* HWP1* gene, we could easily identify and confirm these isolates as* Candida albicans*. Based on our data, the activity rate of hemolysin and the ability to produce biofilm among the isolates play a role in the pathogenicity of the* Candida.* In this study, for the first time, the cohemolytic effect between* Candida* and vaginal microflora was evaluated.* S. aureus* and* S. agalactiae* were CAMP-positive and showed synergy with almost half of the isolates. Overall, these bacteria were found to have a role in increasing pathogenicity of* Candida* in vaginal candidiasis.

## Figures and Tables

**Table 1:** Distribution profile of enzymatic activity, biofilm production, and CAMP-like effect on vaginal *Candida albicans* species isolates. *S. aureus (aur.)*, *S. saprophyticus (sap.)*, and *S. agalactiae* (aga.).

Species	Tests								
	PCR *(HWP1)*	Hemolysin	CAMP-like effect			Biofilm			
		beta	sap.	aur.	aga.	Neg.	1+	2+	3+
*Candida albicans*	110	110	0	48	54	41	28	19	22
Percent	100%	100%	0%	43.6%	49%	37.3%	25.4%	17.3%	20%
